# Regional differences in HIV prevalence among drug users in China: potential for future spread of HIV?

**DOI:** 10.1186/1471-2334-8-108

**Published:** 2008-08-04

**Authors:** Mirjam Kretzschmar, Weidong Zhang, Rafael T Mikolajczyk, Lan Wang, Xinhua Sun, Alexander Kraemer, Fan Lv

**Affiliations:** 1 School of Public Health, University of Bielefeld, Bielefeld, Germany; 2 Centre for Infectious Disease Control, RIVM, Bilthoven, The Netherlands; 3 Julius Centre for Health Sciences and Primary Care, University Medical Centre Utrecht, Utrecht, The Netherlands; 4 National Center for AIDS/STD Control and Prevention, China Center for Disease Control and Prevention, Beijing, PR China; 5 Department of Disease Control, Ministry of Health, Beijing, PR China

## Abstract

**Background:**

Drug use and in particular injecting drug use has been at the forefront of the explosive spread of HIV in general populations in many countries in Asia. There is concern that also in China increased HIV incidence in drug users might spark off a generalized epidemic in the wider population. Close monitoring of HIV incidence and risk factors in drug users is therefore important to be able to target interventions effectively. Second generation surveillance was launched to assess HIV prevalence and risk behaviours jointly with the purpose of describing trends and predicting future developments. To assess whether these goals were fulfilled among drug users in China we provide an analysis of risk factors for HIV infection and of regional differences in HIV prevalence.

**Methods:**

We analysed data collected in 2005 in 21 drug user second generation surveillance sentinel sites from 14 provinces in China. We used random effects logistic regression to test for risk factors for HIV infection and regional differences.

**Results:**

The overall HIV-1 antibody prevalence was 5.4% (279/5128); 4.9% among injecting drug users (IDU) not sharing needles and 3.7% among non-injecting drug users. We found substantial heterogeneity among the surveillance sites with prevalence rates ranging between 0% and 54%. HIV status was strongly affected by the regional prevalence of HIV. Risk behaviours were highly prevalent in regions where HIV prevalence is still low. The distribution of duration of drug use in different sites indicated different stages of the drug use epidemics.

**Conclusion:**

]Regional differences in HIV prevalence in China reflect different stages of the drug use and HIV epidemics rather than differences in risk behaviours. Therefore, outbreaks of HIV among drug users in regions where prevalence is still low can be expected in the future. However, methodological limitations of surveillance embedded into routine systems limit the usability of existing data. More standardized approaches to data collection in secondary generation HIV surveillance are necessary to better understand regional differences in risk behaviour and prevalence and to design targeted intervention for those regions at risk of experiencing outbreaks.

## Background

The first recognized outbreak of HIV in China occurred in 1989 in Ruili City, Yunnan Province, among injecting drug users (IDU)[1]. In the years to follow HIV spread explosively among IDU populations in Yunnan province and subsequently in neighbouring provinces. In 1995 a sentinel surveillance system was initiated, the so-called first generation surveillance. Only in 2001 the Chinese government acknowledged the existence of a major epidemic and changed its course to more openness and proactive behaviour to control the increasing HIV/AIDS problem [2]. With international support from the World Health Organization (WHO) and the Joint United Nations Programme on HIV/AIDS (UNAIDS), a first published estimate of the size of the problem was derived combining estimates of the sizes of risk populations in the various provinces with estimates of HIV prevalence per risk group available from national surveillance sites in 2003. The main risk groups considered in this estimation were IDU, men who have sex with men (MSM), paid blood donors infected by unsafe equipment, and commercial sex workers (CSW). It was estimated that there were between 650,000 and 1.2 million people living with HIV in China in 2003. Newer estimates tend to be somewhat lower and put the number of people living with HIV in 2005 between 540,000 and 760,000, the number of new HIV infections in 2005 at 70,000 to 80,000 and 25,000 AIDS deaths [3]. There is substantial regional heterogeneity in HIV prevalence with those provinces bordering to the Golden Triangle, where drug trafficking is common, having the highest HIV prevalences, especially among IDU. In the prefectures of Yunnan and Xinjiang prevalences of up to 80% among IDU have been reported [4,5]. Also in the provinces of Sichuan, Guizhou and Guangxi prevalences of around 50% among IDU have been reported [6].

In recent years the Chinese government has intensified its surveillance programme [7], which consists of: a) case reporting through an internet based surveillance tool, b) sentinel sites where mainly prevalence data is collected, and c) second generation surveillance sites where prevalence and behavioural data are collected, d) in depth epidemiological investigations [8]. The sentinel surveillance has been expanded to include 194 sites in 2003, of which 49 are drug-user sites from 21 provinces. Following recommendations by WHO for implementing second generation surveillance [9], another set of surveillance sites was established specifically for collecting behavioural data aimed at monitoring trends in risk behaviour and the prevalence of other sexually transmitted infections (STI) such as syphilis. At present, there are 105 sites for second generation surveillance. The sites were chosen according to numbers of case reports in the national surveillance and other epidemiological considerations. Concerning drug users, provinces with HIV prevalence above 5% have to have at least one sentinel surveillance site collecting behavioural information; in other provinces sites can be chosen based on factors like local socio-economic level, local health care facilities, and the size of floating populations. In 2005, 21 sites in 14 provinces collected behavioural data in drug users.

Persons identified as drug users in China are registered in a database of the Ministry of Public Security. The numbers of registered drug users have risen from 70,000 in 1989 to 1,140,000 in 2004 [10]. It was also estimated that the total population of drug users in 2004 was around 3.5 million and thus about three times as large as the number of registered drug users [11,12]. It is estimated that among drug users about 54% are injecting drug users and of those about 45% have recently shared needles and other injecting equipment [13,14]. Large regional differences exist, however, with the highest rates of injecting and needle sharing observed in the southwest provinces and Xinjiang. Also, it has been shown that a correlation exists between high risk injecting and high risk sexual behaviour in male injecting drug users [15].

Although up to now the epidemic in China has remained concentrated in risk groups, there is increasing concern that HIV might spread via bridge populations such as female sex workers [16,17] or surplus men [18] into the general population. Many female IDU are also CSWs and commonly engage in unprotected sex [19]. Recently, a molecular epidemiological study has shown increased circulation of sexually transmitted HIV in Yunnan, the province with the first and largest HIV epidemic among IDU [20].

In view of the imminent threat of large scale spread of HIV into the general population of China a surveillance system that allows reliable monitoring of the epidemic and of possible changes in risk behaviour is important. Second generation surveillance of drug users was implemented in 1999 and since then scaled up to the present dimension. Here we analyse data from second generation surveillance among drug users conducted in 2005 with data collected in 21 drug user sentinel sites from 14 provinces. While most studies have focussed on the HIV epidemic in southwest China [14] we report on data from sites all over the country. In this analysis we focus on the geographical differences of HIV prevalence and drug use. The prevalence of syphilis and implications for the further sexual spread of HIV will be analyzed elsewhere. Central aims of second generation HIV surveillance are focussing surveillance on subgroups of the population at highest risk of infection and implementing surveillance that moves with the needs and state of the epidemic [21]. We present results that contribute to assessing whether those aims can be reached with the SGS system as implemented in China at present and what the main limitations of the collected data are in this respect.

## Methods

### Choice of second generation surveillance sites and study population

In 2005, based on the HIV prevalence obtained from the HIV/AIDS case reporting system in 2004 and other epidemiological studies, 22 drug user sites from 14 provinces were initially selected to perform a behavioural survey. Each site was asked to recruit between 300 and 400 participants. This number was considered sufficient for descriptive purposes and to estimate prevalence with 95% confidence intervals of +/- 5% (for prevalence rates below 30%). Persons to be included were drug users who smoke or intravenously or intramuscularly use heroin, cocaine, opium, marijuana, morphine, methamphetamine hydrochloride, Pethidine, K powder (Ketamine), or Methylenedioxymethamphetamine (MDMA, also known as Ecstasy). In order to avoid prosecution by law and increase participation, anonymity and confidentiality were guaranteed to the drug users when they participated in the survey. Recruitment followed convenience sampling: some participants were recruited from detoxification centres, others in public meeting places where drugs are consumed, and finally in bars and outreach projects. In some cases snowball sampling was also used. The proportions recruited from these settings differed between different sites due to different local circumstances.

The data collection period was from July to September 2005, but could be prolonged in case a site was not able to recruit the requested number of participants within that time period. If the required sample size was not reached by the end of the year, local investigators contacted China Center for Disease Control and Prevention who then decided whether or not to end the sampling process. All participants were asked to complete a questionnaire on demographic and behavioural issues and to provide a blood specimen for HIV antibody testing. One site in Kunming, Yunnan province was excluded from our analysis because information about risk behaviour of drug users was missing. The protocol "HIV Surveillance Protocol at National Comprehensive (second generation surveillance) HIV Sentinel Sites", was approved by the national ethical committee of China [7]. The protocol requires informed consent from participants in behavioural surveillance.

### HIV testing

The blood specimens were collected on-site and tested at the provincial CDC. Two Enzyme-linked Immunosorbent Assays (ELISA) were used for testing for HIV antibodies. For blood specimen with a positive result in the first ELISA, a second (different) ELISA assay was performed (first HIV ELISA test: ELISA reagent: (Wantai Biotech, Beijing, China); second HIV ELISA test: ELISA reagent (Vironostika HIV Uniform II Ag/Ab. Akzo Organon Teknika, Netherlands). Specimens with positive results in both ELISA assays were considered positive for HIV.

### Behavioural survey

Information asked in the questionnaire included recruitment setting, gender, age, marital status, place of residence, ethnicity, educational background, profession, monthly income, age at first drug use, injecting drug use and needle sharing, number of commercial sex partners in last 12 months, and STI symptoms in last 12 months. In addition to the behavioural risk factors, knowledge about transmission routes of HIV (six questions: "can HIV be recognized from appearance?"; "can HIV be transmitted by blood products?"; "by needle sharing?"; "from mother to child?"; "can transmission be prevented by condoms?", "by selecting uninfected partners?") and participation in prevention programs (needle exchange, methadone replacement, partner education, condom distribution, HIV counseling and testing, and receipt of AIDS/STI brochures) were assessed. Both sets of variables were dichotomized: all answers correct versus other and receipt of any intervention versus no intervention. We collected information about injecting drug use and needle sharing using different recall time intervals. For the analysis reported here we used only the information about ever injecting drugs and ever sharing needles.

### Statistical analysis

During data cleaning 649 records were removed. In 621 cases no blood test was performed (blood samples were either insufficient for conducting the HIV test, blood taking was not successful or the participants did not comply with taking a blood sample). Respondents reporting first drug use at age below 14 years (27 cases) were excluded. When the fraction of missing responses for a specific variable was larger than 5% missing responses were coded as a separate category. The final sample size after data cleaning and exclusion of participants with missing responses (for missing values < 5%) was 5128. We assessed the effects of missing values on the results by repeating the analysis with separate categories for missing values (N = 5593).

A Chi-square test with significance level p ≤ 0.05 was used for evaluation of bivariate associations between socio-demographic and behavioural risk factors and HIV status. A joint analysis of all considered risk factors was carried out using random effects multiple logistic regression [22]. First, an analysis including all variables was performed with site as a grouping factor but without any covariates at the site level. Second, we investigated whether the observed regional heterogeneity could be explained by differences in the prevalence of HIV between regions. We generated a variable containing the information about prevalence in each region and included this as a site level covariate in the model. Finally, differences between different study sites were presented as frequencies of the respondents with different characteristics using the sample of 5890 participants with available information. The distribution of duration of drug use was analysed to assess the composition of the regional samples and the epidemiological situation of drug use in the regions.

## Results

### Characteristics of the total sample

In total, 6539 interviews were conducted in 21 surveillance sites and 5918 blood specimens were tested. About half of the respondents originated from detoxification centres, one third from the community, and the remainder from other recruitment settings (Table 1). The sample was predominantly male with 5.6:1 male-to-female ratio. Age range was between 14 and 78 years, with a median of 30 years. Eighty percent of the participants initiated drug use before age of 30 years; about half of the sample had never injected drugs. Among the 2717 IDU 44.6% had ever shared needles. Eighty seven percent of the respondents had no commercial sex partners in last 12 months before the interview, 2% had at least one sex partner with a large variation in numbers, and for 11% the information was missing. Among 85 respondents who had commercial sex, 36.8% had used a condom during the last commercial sex (not shown). In the 12 months before the interview 14% of participants had experienced symptoms that could be caused by STI.

**Table 1:** General characteristics of the study population (N = 5128), their bivariate association with HIV-1 antibody status, and results from random effects multiple regression model.

Variables		Number	%	*HIV*^+^	p*	OR (95% CI)	*p*
Sample source	Detoxification center	2871	56.0	5.8	0.002	1	
	Community	1621	31.6	4.0		0.77 (0.55–1.09)	0.143
	Others^1^	636	12.4	7.6		0.66 (0.46–0.93)	0.019
Gender	Male	4348	84.8	5.5	0.940	1	
	Female	780	15.2	5.4		0.73 (0.50–1.07)	0.111
Age	<= 30	2676	52.2	6.2	0.012	1.04 (0.80–1.36)	0.750
	> 30	2452	47.8	4.6		1	
Marital status	live without partner	2821	55.0	5.1	0.195	1	
	live with partner	2307	45.0	5.9		1.00 (0.79–1.27)	0.991
Place of residence	Local	4393	85.7	5.9	< 0.001	1	
	Transient	735	14.3	2.5		1.27 (0.64–2.51)	0.490
Nation	Han	3794	74.0	2.9	< 0.001	1	
	Minority	1334	26.0	12.7		1.38 (0.93–2.04)	0.107
Education	<= 9 years	1710	33.4	9.0	< 0.001	1	
	> 9 years	3418	66.6	3.7		1.17 (0.89–1.53)	0.250
Profession	white collar	1744	34.0	8.6	< 0.001	1.18 (0.85–1.64)	0.313
	blue collar	3384	66.0	3.8		1	
Monthly income	<= 600	2147	41.9	6.9	< 0.001	1	
	601–2000	1127	22.0	4.5		1.56 (1.13–2.15)	0.007
	> 2000	313	6.1	1.9		0.51 (0.24–1.08)	0.080
	Missing	1541	30.1	4.7		1.05 (0.79–1.39)	0.751
Age at first drug use	<= 30	4189	81.7	5.5	0.05	1	
	> 30	707	13.8	4.1		0.71 (0.48–1.05)	0.088
	Missing	232	4.5	1.6		0.79 (0.38–1.65)	0.532
IDU and sharing needles (ever)	no IDU	2411	47.0	3.7	< 0.001	1	
	IDU, no sharing of needles	1506	29.4	4.9		1.89 (1.37–2.61)	< 0.001
	IDU, sharing needles	1211	23.6	9.5		3.15 (2.29–4.33)	< 0.001
Having commercial sex in last 12 months	No	4455	86.9	5.5	0.148	1	
	Yes	85	1.7	9.4		1.33 (0.66–2.70)	0.4265
	Missing information	588	11.5	4.4		0.73 (0.50–1.06)	0.102
STD symptoms in last 12 months	No	4418	86.2	5.3	0.338	1	
	Yes	710	13.9	6.2		0.76 (0.55–1.05)	0.100

^1^Outreach programs, contacts of known IDU, unknown.

p* Chi-Square

### Factors associated with HIV in the total sample

The overall HIV-1 antibody prevalence was 5.0%; 6.6% among IDU and 3.5% among non-injecting drug users. Bivariate analysis showed several associations between the investigated variables and HIV-1 antibody status. HIV prevalence was higher in participants who: were recruited in detoxifications centres; were younger (≤ 30 years), were resident versus transient; were from minority ethnic groups versus Han-Chinese; had higher versus lower education; had 'blue collar' versus 'white collar' jobs, had higher incomes; were younger at first drug use; had ever injected drugs; and had ever shared needles (Table 1, columns 3 and 4).

Random effects multiple logistic regression analysis showed fewer significant associations: income categories, ever injecting drugs, ever sharing needles and recruitment setting were associated with HIV status (Table 1, columns 5 and 6). Most prominent were the effects of ever injecting drug use (OR = 1.9) without sharing needles or with sharing needles (OR = 3.2) as compared to non-injecting drug use. Participants in the middle income category had highest risk for being HIV positive. The analysis with all missing values recoded into separate categories yielded very similar results (data not shown).

Overall, the impact of regional differences (as random effects) was large, explaining 87% of the variation in the sample. However, the heterogeneity across sites was substantially reduced after adjusting for regional differences in HIV prevalence (explaining 62% of the total variation). At the same time, the model prediction (and the strength of the association between risk factors and HIV status) considerably improved (area under the receiving operator curve increased from 0.78 to 0.89). Since injecting drugs (with and without needle sharing) was the most prominent risk factor, only the interaction between this variable and regional prevalence was investigated and there was no statistical evidence of interaction (p > 0.05).

### Regional diversity in the prevalence of HIV and in risk behaviours

**Figure 1:**
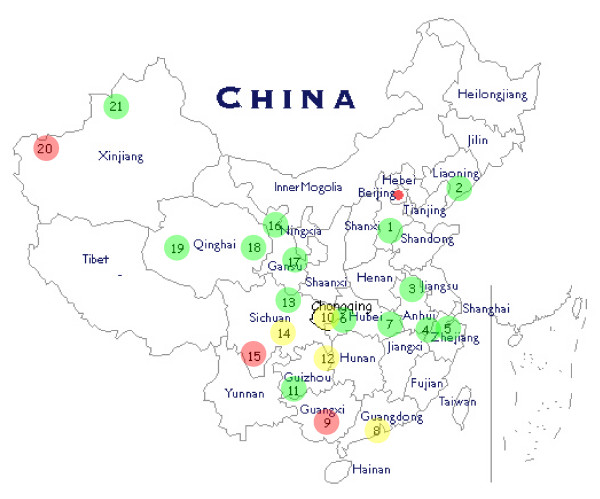
**Sentinel sites for second generation surveillance among drug users in 2005, in brackets the HIV-1 prevalence.** Green dots mark sites with HIV prevalence < 1%, yellow dots sites with HIV prevalence between 1% and 10%, and red dots sites with HIV prevalence > 10%. 1 Hebei Shijiazhuang (0%). 2 Liaoning Fushun (0%). 3 Anhui Huainan (0%). 4 Anhui Maanshan (0.7%). 5 Zhejiang Hangzhou (0%). 6 Hubei Jingzhou (0.5%). 7 Hubei Xiaogan (0.9%). 8 Guangdong Dongguan (5.9%). 9 Guangxi Nanning (21.0%). 10 Chongqing (7.3%). 11 Guizhou Qianxinan (0.4%). 12 Guizhou Tongren (4.5%). 13 Sichuan Dazhou (0.4%). 14 Sichuan Leshan (2.6%). 15 Sichuan Liangshan (29.4%). 16 Gansu Dingxi (0%). 17 Gansu Wuwei (0%)). 18 Qinghai Haidong (0.6%. 19 Qinghai Haixi (0%). 20 Xinjiang Kashi (10.8%). 21 Xinjiang Yili (0%).

There was a substantial heterogeneity among the surveillance sites (Table 2). The HIV prevalence was higher in three regions (21% in Guangxi Nanning, 29% in Sichuan Liangshan and 10.8% in Xinjinag Kashi), up to 8% in 10 regions and no HIV cases were found in samples from 8 sites (Figure 1). The fraction of participants recruited in detoxifications centres varied between 0% and 100%, the proportion of participants who had never injected drugs varied between 2% and 98%. On the other hand, between 0.3% and 71% were drug users who had injected drugs and shared needles. Information regarding the number of commercial sex partners in the last 12 months was missing for a substantial fraction of respondents making the assessment of exposure difficult. There were also large differences in the proportions of participants tested in the past for HIV (7%–57%) and who participated in any drug use and HIV related interventions (0%–75%). There was also a substantial difference in the fraction of participants who knew all the correct answers to questions related to HIV ranging from 30% to 80%. Also, in terms of demographic aspects the samples strongly differed with the proportion of female participants ranging from 0% to 49% and the proportion of people from minority ethnic groups from 0% to 98%. There was no clear pattern of differences using cluster analysis; the only correlation between the variables was found for the fraction of respondents tested in the past for HIV and the fraction who participated in any intervention in the past, which is not surprising.

**Table 2:** Prevalence and risk factors for HIV infection among drug users in all surveillance sites.

	Sites	Total	HIV prevalence % (95% CI)	From detoxification center %	IDU and needle-sharing – ever (%)		Number of commercial sex partners in last 12 months (%)			Tested for HIV in the past %	Participated in any intervention %	Knows all answers %	Female %	Ethnic minority %
										
					IDU without needle-sharing	IDU with needle-sharing	None	Any	Missing					
1	Hebei Shijiazhuang	361	0.0 (0.0–1.1)	13.0	47.1	19.8	95.0	0.3	4.7	24.1	17.7	65.1	18.8	3.5
2	Liaoning Fushun	28	0.0 (0.0–12.1)	26.7	25.0	71.4	92.9	0.0	7.1	39.3	35.7	50.0	0	0
3	Anhui Huainan	113	0.0 (0.0–3.2)	0	33.6	19.5	89.4	0.9	9.7	51.3	0	65.5	28.3	2.7
4	Anhui Maanshan	141	0.7 (0.1–3.9)	0	65.0	19.3	92.9	0.7	6.4	36.2	9.9	80.1	33.3	3.6
5	Zhejiang Hangzhou	267	0.0 (0.0–1.4)	99.3	32.8	20.1	91.8	0.4	7.9	22.5	39.0	68.9	29.6	4.5
6	Hubei Jingzhou	409	0.5 (0.1–1.8)	98.1	41.9	28.6	89.2	2.9	7.8	21.3	26.2	79.5	14.4	0.2
7	Hubei Xiaogan	352	0.9 (0.3–2.5)	72.1	37.7	19.1	77.0	0.3	22.7	7.4	66.2	74.7	27.3	2.9
8	Guangdong Dongguan	407	5.9 (4.0–8.6)	39.2	44.8	21.9	76.9	4.4	18.7	57.0	11.1	37.3	9.8	11.8
9	Guangxi Nanning	291	21.0 (16.7–26.0)	53.2	44.0	53.6	90.0	2.1	7.9	12.4	75.3	74.2	49.1	17.3
10	Chongqing	368	7.3 (5.1–10.5)	63.7	38.8	52.2	77.4	1.1	21.5	37.2	28.5	62.5	0	.3
11	Guizhou Qianxinan	363	0.3 (0.1–1.6)	99.8	32.6	22.1	74.7	2.5	22.9	27.5	3.6	60.1	18.7	19.8
12	Guizhou Tongren	376	4.5 (2.8–7.1)	49.9	30.6	26.9	72.3	1.3	26.3	19.7	37.2	30.3	27.1	53.3
13	Sichuan Dazhou	223	0.4 (0.1–2.5)	50.0	58.7	33.0	73.1	4.9	22.0	28.3	7.6	57.4	20.6	.5
14	Sichuan Leshan	189	2.6 (1.1–6.1)	56.5	14.9	61.2	98.4	0.5	1.1	41.3	69.3	51.3	15.9	2.8
15	Sichuan Liangshan	415	29.4 (25.2–34.0)	76.7	15.4	20.8	90.1	1.9	8.0	26.7	12.0	49.6	6.7	94.9
16	Gansu Dingxi	193	0.0 (0.0–2.0)	64.5	1.0	1.0	100.0	0.0	0.0	38.9	6.2	42.5	3.6	3.2
17	Gansu Wuwei	357	0.0 (0.0–1.1)	44.6	1.4	0.3	98.3	0.3	1.4	34.2	1.7	56.3	3.6	12.0
18	Qinghai Haidong	163	0.6 (0.1–3.4)	0	8.1	21.2	96.9	0.0	3.1	10.4	62.0	40.5	7.4	49.1
19	Qinghai Haixi	478	0.0 (0.0–0.8)	62.6	3.6	2.1	95.6	0.4	4.0	50.6	11.3	60.3	3.8	35.4
20	Xinjiang Kashi	297	10.8 (7.7–14.8)	17.2	12.5	5.1	94.9	3.4	1.7	23.9	14.8	38.0	3.4	98.3
21	Xinjiang Yili	99	0.0 (0.0–3.7)	56.9	46.9	34.7	92.9	0.0	7.1	22.2	41.4	72.7	0	98.0

### Distribution of the duration of drug use as an indicator of epidemiologic stage

**Figure 2:**
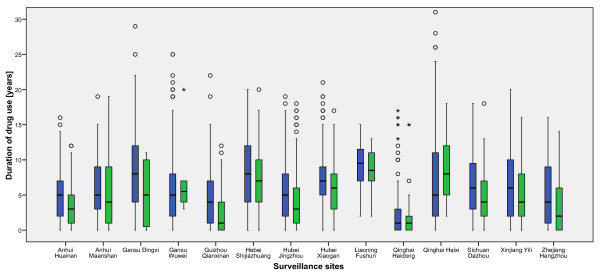
The distribution of the duration of drug use (age of respondent minus age at first drug use) in sites with low HIV prevalence (< 1%) (Blue boxplots – any drug use, green – injecting drug use among IDU).

**Figure 3:**
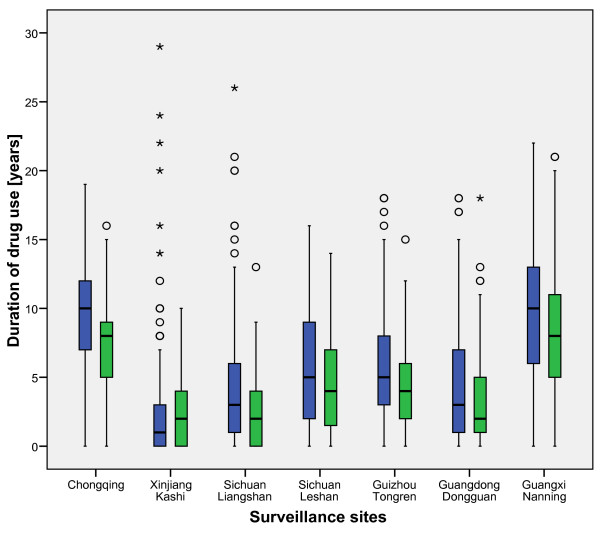
The distribution of the duration of drug use (age of respondent minus age at first drug use) in sites with medium and high HIV prevalence (≥ 1%) (Blue boxplots – any drug use, green – injecting drug use among IDU).

There were substantial differences with respect to the duration of either any drug use or injecting drug use across the sites (Figures 2 and 3). In several sites the median duration was short (below 5 years) but in a few sites it was substantially longer (approaching 10 years). The distribution of the duration of injecting drug use in most sites followed the duration of any use, but the medians were usually lower.

## Discussion

The prevalence of HIV differed markedly between different second generation surveillance sites, ranging from 0% in eight sites to above 20% in two sites (21% in Guangxi Nanning and 29.4% in Sichuan Liangshan). Both of the high prevalence sites lie in provinces bordering Yunnan province – the area where the HIV epidemic among IDU in China first started – or Myanmar. The site in Kunming, Yunnan, which was excluded from the analysis, had a prevalence of 54.0%. Another site with relatively high prevalence was Xinjiang Kashi with 10.8%, which is in the northwest near the border with Afghanistan. In previous years the prevalence in this site was found to be as high as 60% but in 2005 only some of the respondents were actually tested for HIV, so the estimate of 10.8% is probably an underestimate of the true prevalence. These results are similar to the regional distribution of HIV prevalence in China found in earlier surveillance data [3].

Whereas known risk factors like injecting drugs or sharing needles were associated with HIV infection at the individual level in logistic regression, they were not associated with HIV prevalence at the population level. On the contrary, the uncertainty in relating risk behaviour to being infected was greatly reduced when local HIV prevalence was taken into account in the model. This can be seen as an indication of the different phases of the HIV epidemic in different geographical regions, with some regions already heavily affected and others still at the beginning of a large outbreak. Risk behaviours like needle sharing were highly prevalent in many regions, including those with low HIV prevalence, indicating the potential for HIV spread in as yet unaffected regions. Interestingly, the timing of drug use and HIV epidemics differ between sites with some having a well established (injecting) drug use epidemic with low or high HIV prevalence and others where (injecting) drug use and possibly HIV are newly emerging (Figures 2 and 3). In view of the fact that drug users at the beginning of their injecting career are at increased risk of contracting blood borne infections [23-25] the distribution of exposure times can provide important information about the potential for future expansion of the HIV epidemic and for targeting interventions. Interventions were not associated with risk reduction or with a higher knowledge regarding HIV at the regional level. Judging from the distributions of drug use duration in different sites we conclude that sites with mature drug use epidemics lie in the south of the country around Yunnan province, then there is a band with sites where both long term and new drug users are found in the samples, and finally there are some sites with mainly new drug users.

Participants were asked whether they were tested in the past for HIV, but not what their results were. Since a positive test result could have been associated with behavioural changes, we did not include current risk behaviour in the risk analysis model. For the same reason the assessment of current risk behaviour in a regional sample with high HIV prevalence could be biased. Therefore, in the analysis of risk factors for HIV we only used the information on ever injecting drugs and ever sharing needles. On the other hand, since there was no assessment of sexual risk behaviour in terms of ever/never, we used the information about the last 12 months. Clearly, this information could be misleading if behavioural changes followed a positive HIV test. The difficulty could be remedied if the questions about having been previously tested for HIV were complemented by asking about time and result of the test. Also having symptoms of sexually transmitted infections in the last 12 months cannot be simply interpreted as a proxy for a behavioural risk factor because these might facilitate the transmission of HIV infection.

A major limitation of the data set is the lack of a systematic sampling frame across the different sites. The marked heterogeneity in recruitment settings, with some samples coming mainly from detoxification centres and others from the drug user community makes any comparison between sites questionable. In particular, if detoxification is enforced by law as reported in [14] the selection of survey participants would be strongly biased. The representativeness of community-based samples is also unknown, although in some settings results obtained from convenience sampling or even snowball sampling can be unbiased [26,27].

Sampling from different subgroups of the drug user population might influence the distribution of duration of drug use. This might explain the striking difference in HIV prevalence between the two sites located in Xinjiang province. Also, the high HIV prevalence in Xinjiang Kashi among drug users who had just started their drug using career could have resulted from a sampling bias. Problems with lack of systematic sampling frames for drug users have been discussed extensively in the literature and the inherent difficulties have to be acknowledged [27]. Drug use is illegal in nearly all countries and stigmatised in most societies [28] so drug users are likely to hide their activities and avoid contacts associated with disclosure of drug use. In order to reduce these kinds of behaviours and increase participation in the study, our data collection procedures ensured that there was no threat of prosecution. Difficulties with reporting of illegal behaviour might also extend to other areas where some responses are socially desirable, resulting in missing values and untruthful responses. This might have occurred in this survey with the reporting of sexual behaviour. Several methods have been proposed for controlling social desirability bias and should be included in further studies of drug users [29].

Recently, the so-called 'respondent driven sampling' method has been developed to ensure access to drug users who cannot be reached by prevention or health care workers [30,31]. Respondent driven sampling offers the opportunity to develop quantitative estimates of HIV prevalence based on the sample obtained with a sampling frame following strict rules in participant recruitment. However, the method is still being developed and its reliability has to be further investigated [32].

Second generation surveillance for injecting drug users should be further enhanced by surveys assessing knowledge and attitudes about risk behaviour, population size estimates for hidden populations, and hepatitis C surveillance [33]. While questions about knowledge of risk behaviours are already part of second generation surveillance in China, monitoring of hepatitis C could be a valuable addition. As the prevalence of hepatitis C in beginning injectors is seen as a proxy for incidence of new infections, changes in young injectors' hepatitis C prevalence can indicate changes in injecting risk behaviour.

## Conclusion

In conclusion, there are two main findings of this study. First, risk behaviours are prevalent among drug users in China and provide substantial potential for HIV spread in not yet affected regions. Second, methodological limitations of surveillance embedded into routine systems limit the usability of existing data. One of the goals of second generation surveillance according to WHO guidelines is the "better use of surveillance data to increase understanding and to plan prevention and care" [21]. Only when surveillance data lead to an understanding of the epidemic process can effective interventions be implemented. To reach that goal it is advisable that more care be taken to ensure standardized data collection. More capacity and support should be given to surveillance sites to make the investment into second generation surveillance worth the effort.

## Competing interests

The authors declare that they have no competing interests.

## Authors' contributions

MK and RTM designed and conducted the analysis, MK wrote the final version of the text. WZ performed data analysis and drafted the first version of the paper. AK laid contact with China CDC and commented on the manuscript. XS, LW, and FL participated in the design of the second generation surveillance in China and helped to develop the questionnaire; LW collected the raw data from all sentinel sites; FL commented and revised manuscript draft. All authors read and approved the final manuscript.

## Pre-publication history

The pre-publication history for this paper can be accessed here:


